# Epidemic Cholera in Yucatan

**Published:** 1833

**Authors:** Henry Perrine

**Affiliations:** U. S. Consul


### WESTERN JOURNAL

OF THE

MEDICAL AND PHYSICAL SCIENCES.

OCTOBER, NOVEMBER AND DECEMBER, 1833.

### Essays and Cases.

### Art. I.—Epidemic Cholera in Yucatan.

*An account of the Epidemic Cholera, in Campeche, and other parts of Yucatan, in a letter from* Dr. Henry Perrine, *U. S. Consul, to the Editor.*

*Consulate, U. S. A. Campeche, 20th Sept.* 1833.

Dear Sir,

To-day for the first time, I have read your account of the Cholera in Cincinnati last fall. The only medical periodical of the United States which I have seen since my departure from New-York in Oct. last, are the 21st and 23d Numbers of the Philadelphia Journal of the Medical Sciences, which arrived ten days ago. You perceive, therefore, that I could not have been influenced in my treatment, by the reports of your physicians, as the Asiatic, Malignant Cholera entered Campeche, *officially*, on the 24th June, and ceased, as an epidemic, on the 21st July.

You talk about the frightful mortality it occasioned during six weeks in Cincinnati. If the truth were known, I believe that Campeche, with a less population, would be found to have lost as many inhabitants in a single day, as you lost in a month. *Officially* we buried only about four thousand in four weeks, and not more than four hundred on the most fatal day. Estimating the whole population remaining in Campeche at 20,000, the mortality was nominally but twenty per cent. In a village only five miles distant, along the coast, where there was no medical assistance, at least 40 per cent of the original number perished, but as the greater proportion fled at the beginning, you can estimate how dreadful was the havoc among those that remained. Some villages and plantations have been entirely depopulated—while others have been very slightly affected!

The regular rains set in very late at Campeche, but as soon as they commenced, the epidemic began to decline. It then extended to Merida, where it appeared with much less malignity, but endured more than double the time. In Campeche it indeed spread with the violence of a hurricane. The buildings of the city and suburbs extend above three miles along the sea shore, and are bounded on the southeast and west by a semicircular range of hills, all of cavernous limestone. The city proper is surrounded by high walls, having four gates opening at the cardinal points, and is fortified with cannon, and garrisoned by soldiers. But when the Cholera came, the sentinels dropped down at their posts, and the guards at the gates—which were then left open the whole night long.

Priests fell in the procession, while carrying the Saint or the Lord whose intercession they invoked. Devotees were attacked on their knees at the Altar, while soliciting the favor of the Virgin Mary. Candles burning before the images in their houses, pious pictures strewed on their beds—holy wooden heads and wooden hands on their pillows and on their breasts—all were of no avail. The houses were soon all closed, and the streets all deserted, except by those whom necessity forced to seek food, medicines or coffins, the Doctor, the Priest, or the dead-cart—sometimes all together. Very soon, coffins ceased to be made, and graves to be dug. An armed force alone, could compel assistance for the dying or the dead of the hospitals. Propositions to burn the bodies, were substituted by shallow excavations in the sand of the sea shore, as a less laborious measure. The corpses of the most respectable persons were slung in net-hammocks to a pole, and carried by two men, to some separate hole which they scraped among the bushes.

The mass of dead bodies were piled up in loads like logs, and carted to the common ditch on the shore, where they were laid in rows and covered with a few shovelsfull of sand. The skeletons of several who attempted to fly, were found divested of flesh by beasts or birds of prey. A child apparently a month old, was found alive on the road side, but no traces of the mother were ever seen. Seven priests followed their flocks to unconsecrated graves. Famine accompanied the plague. Provisions advanced three or four hundred per cent. Crowds collected at the doors where corn was to be sold, but many were carried off by the Cholera before it came to their turn to receive a handkerchief full. Finally the living began to envy the dead. Happy the dead, was the general remark, they had some aid during life, something like interment after death—but we must perish alone and be consumed by dogs and buzzards in our dwellings.

But I did not begin this letter with the intention of troubling you with my undigested recollections of the Cholera in Campeche. These I have promised to Doctor Dewees, as soon as my health and occupations will admit my placing them together. My especial object was to thank you for the testimony which you have borne to the merits of Calomel in the treatment of this disease, and to refer you to my own experience in its favor. So strong and sincere was my conviction of its great curative powers, that on the 9th July, during the height of the Cholera, I addressed as long a letter as the times would admit, to Doctor Hubble of Merida. On the 13th July, I recommended the calomel treatment, in a letter by express, to the Governor of Yucatan; and on the 15th, I deemed it my duty to record the substance of my experience, in the form of an official letter, to the Secretary of State of the U. S. A. Copies of the last two or official letters, I have forwarded to Doctor Dewees of Philadelphia: but even these do not express all the confidence which I now feel in the treatment of Cholera by *calomel alone.*

You must keep in view, that the Cholera of Campeche was the genuine Cholera of India, not the Cholera of Paris nor of New York. It had no *stage* of premonition, of reaction, of collapse. It was all one *exhausting march,* from the beginning to the end, to the exhaustion of death. The exceptions were but exceptions. I should as soon think of talking about the *stages* of reaction, or of collapse, in uterine hemorrhage, as in Campeche Cholera. Whether the torrent of blood or of water from the vessels, appear externally or remain internally, the result is the same—the patient continues sinking, wasting, exhausting till he dies! The flow of serum from the surface of the intestinal tube into its cavities, whether vomited or purged out of them or not, will drain away from the system, and sink the pulse, heat and life, as fast as a flow of blood. I should like to see the corpse of a Cholera patient whose bowels are not full of water, if they were not emptied by vomiting or purging during life. One patient will die on the stool, from a continued stream of fluid which is flowing *out of* his bowels, and another in bed, from an equal stream which is flowing *into* his bowels. In both the blood and heat are departing from the surface—both are becoming emaciated, cold and pulseless—but the latter is supposed not to be in as much danger, *because he has no discharges from his bowels!* My views then, you perceive, lead me to check the watery secretion *into the* intestines as soon as possible—whether it be or be not discharged *out of* them—and this serous flooding I am convinced, may be generally suppressed by large doses of calomel. I need not tell the Physicians of the West and South of the U. S. that the larger the dose of this medicine, the less is that ultimate or occasional effect called catharsis, and the longer the time before it appears. I need not inform many of them, that in a *large* dose it will check serous diarrhœa more speedily than opium or alum. I do not use the general terms, narcotics and astringents, because I believe that the classifying titles of remedies is as injurious to reasoning, as the classifying names of diseases. To me they sound like technical slang whose theoretical associations always embarrass the judgement more than they aid the memory. For me it is sufficient to know, that 40 grains of calomel will check serous intestinal secretions—but whether it should hence be classed among the diffusible stimulants which calm irritation, or among the astringents, which contract the exhaling vessels, I neither know nor care. I know that in very many cases, where 40 grains of calomel have rested in the stomach of a choleric patient, that within half an hour his whole surface has become warm, his cramps have ceased, his vomiting and purging have not returned, and he has got well, with rest and toast water. I know that in these very cases, there was no necessity for tormenting the patient with stimulating frictions or plasters, nor with hot bricks, bottles, irons, or baths of either hot air, or hot water; nor with a load of bed clothes. His dose of calomel, his comfortable quantity of clothing, his desirable quantity of desired drinks until his appetite returned, and his rice or arrow root gruel a few days afterwards, constituted all his treatment.

On the 24th June, there were in Campeche, seven licensed practitioners of Medicine—viz. two old Spaniards, one young Spaniard, one young native of Havana, one old Frenchman, and two young Americans—one being a former student of mine, and the other a Physician who came with me from New York last fall. One old Spaniard left the city the first day, the young Spaniard shut himself up the first week, the other old Spaniard and the old Frenchman shut themselves up the second week—the young Havanese died about the same time—and the two Americans, besides myself, continued in the streets during the whole epidemic. *We* all used calomel—with this difference, that my companions generally prescribed twenty grains with one of opium, to be repeated every three hours—while I prescribed forty grains without opium, to be repeated as often as debilitating vomiting or purging occurred, even if it should be as frequently as every five or ten minutes. As the Spaniards and French, know little about calomel, and consequently have horrible fears of its effects, they never used it, until their piddling remedies failed, and then finding that it would not cure *dying* patients, they gave themselves up to despair.

As my services were entirely gratuitous, I distributed them in such a manner as most to favor my desire for observation. I therefore assisted the Havanese in his hospital and other physicians in private practice, during the first three days, to see the effects of their treatment, prescribing myself only for occasional cases in which I could best watch the results of my own views. In this short time, however, the small powders of the two young Americans and the large powders of myself, began to excite public attention—so much so, indeed, that the friends would request me to send the powders, if I could not go myself. I then requested the other physicians to observe the cases treated with forty grain doses of calomel—and finally, on the seventh day (when the interments exceeded three hundred) I deemed it my duty to make the treatment as public as possible. As the procession of St. John of God, on Thursday, had not arrested the progress of the epidemic, it was determined on Sunday to try the powerful influence of the wonderful Lord of St. Roman, (the name of the Western suburbs in which the Cholera broke out, a few days after the return of a vessel from Tampico, with sick aboard.) The Senor de St. Roman was therefore brought with extraordinary splendor, from his church in the suburbs, to the Cathedral in the city. The authorities of all classes vied with each other in being his immediate servants and followers, and the streets were crowded with all the people that had strength enough to leave their sick relatives and friends. As the Lord was about leaving the Cathedral, I drove up into the public square—called out to all the members of the City Councils that I could see—and carried them with me to the City Hall. I bade one take the seat of the Mayor, and thus began, 'Gentlemen, I have not yet seen a patient die who has retained forty grains of calomel in his stomach an hour, if administered while heat and pulse were still present, &c." The result was, to order the printing and distribution of advertisements, the making and distribution of calomel powders—with the directions to take and repeat forty grains, until one dose should remain in the stomach or the doctor should arrive. The commissioners in each ward of the city and suburbs, and the sergeants in each company of soldiers, were furnished with the powders to administer in every case where a physician was not at hand. Some citizens who, at the beginning, would not venture near a patient without a handkerchief sprinkled with camphorated spirits, at their nose, threw away their essences and their fears, and became the curers of Cholera with calomel.

Indeed these valiant volunteers saved more patients, than even we, the Calomel doctors, in proportion to the numbers they attended, as we were called to so many hundreds in a day, that we could rarely arrive at the bedside of any in time.

While the Calomel promised to hold out, when I saw a patient in the cold or pulseless state of exhaustion, I gave him a dose, not with the expectation of curing him; but with the view of relieving his cramps, checking his discharges, and thus enabling him to die with more decency and comfort to himself, and with less labor and distress for his friends. When the Calomel, however, become scarce, and it remained doubtful whether any could be obtained from Merida (40 leagues distant) I reserved the dose for another who should offer chances for recovery. At this period I was requested by a lady, who had lost five servants in succession, to see the sixth who had begun taking the doses of twenty grains of Calomel and one of opium, at three hours apart, but still continued vomiting and purging. Seeing the girl in an apparently desperate condition, I turned to her mistress with a forty grain paper, saying "Madam, it is better to reserve this for the next one that may be attacked in your house; and remember that the cure consists essentially in giving as soon as possible as much Calomel as possible. I have generally found, however, that at the very beginning, if a forty grain dose is retained in the stomach, it is enough, but it is better to err by giving too much than too little." The next day, the lady surprised me by saying that the servant was recovering, that she had given her, after my departure, six doses of forty grains each, till the vomiting ceased, that her bowels were bound, and her kidneys were open, and her mouth very sore. I directed her then to drink barley water with a small quantity of cream of tartar, so as to keep up the current of urine, and secure one daily evacuation from the bowels. She had nothing afterwards but gruel. By the by, I had almost forgot to say that one of the greatest advantages of the Calomel cures, is, the durability of its effects, so that a relapse is scarcely ever known. Under other treatment, one half of the patients nominally cured, died of relapses, or had a lingering convalescence. Those who object to the use of Calomel for fear of a salivation, are unworthy of a serious answer. Admitting that in all cases it should occur and be in proportion to the quantity taken, I say, better a sore mouth than a cold grave. But in the majority of cases, salivation does not occur, and is no more liable to follow five hundred than fifty grains of calomel—if indeed *as* liable as five grains. I have seen much worse mouths after very small than very large doses of calomel—the latter operating most generally on the skin or the kidneys or both. In a killing disease like the Cholera, individuals must risk the peculiarities of their constitutions. When any of my convalescents, to whose mouths the Calomel had been invited by scorbutic gums, rotten teeth, or catarrhal fluxions of the cheeks, complained of the soreness or ulceration of their mouths, I relieved them by asking if they were acquainted with any in the same condition, who had been carried to the "Holy Field" or Campo Santo. But to return to large quantities of Calomel. On the 20th of July, at night, I went to the College of St. Joseph, to stay with some convalescing Priests. They carried me into a room, where lay a servant, who having been attacked in the morning, had taken during the day, three doses of twenty grains of calomel and one of opium, but had vomited all. His skin was as cold as marble—he had not the slightest trace of pulse—his cramps were very violent—he was very thirsty, and had that restlessness which generally betokens the approach of death. I therefore told them to let him die in the cool net hammock in which he desired to be, but at the same time related what the lady had done to her female servant, when I had refused to prescribe. As he had no pulse at all, he was considered a still more legitimate subject for experiment. He was allowed to drink as much cold water as he pleased, and by midnight had taken twelve doses of calomel of forty grains each, after which, he vomited and purged no more. His pulse did not return till twelve hours afterwards, but without any thing else he got well and so remains at this date.

As this first case of recovery from pulseless Cholera, was the last pulseless case that I saw, I cannot, of course, say more about it than this, that it will encourage me hereafter to use large doses of Calomel in those desperate cases, which have heretofore been considered beyond the reach of every remedy and of all combined. In writing this to you, I take it for granted, that you have seen or will see the copies of my letters to our Department of State, and to the Governor of Yucatan, which I forwarded to Dr. Dewees. So difficult it is to make our northern physicians believe that medicines do not follow arithmetical rules, that calling ten grains of Calomel a cathartic, they infer, that sixty grains must produce six times as great a cathartic effect. Doctor C. S. an old respectable physician of New Brunswick, New Jersey, was endeavoring to check the Choleric discharges from the bowels of one of my early friends, one night in August, of the past year, by the same doses of laudanum which had proved ineffectual during the preceding day. As I was alarmed for his safety, I energetically insisted on the doctor's giving him a drachm of Calomel, assuring him that the ultimate cathartic effect of the Calomel, would only show itself late the ensuing day, if at all, in the shape of tea leaves, tar, or mud, while its primary and immediate effects, would be those he was seeking to obtain by *narcotics* and *astringents*. As the already secreted contents of the intestines, however, continued to be discharged in small quantities, during the first few hours after taking the Calomel, the doctor triumphantly pointed them out to me, as effects of the large dose of Calomel! I could only reply, that the same logic would prove large doses of opium, to be purgative, in all cases were they do not immediately check a diarrhœa. A similar difficulty I encounter with similar doctors, in making them believe that ten grains of Quinine will reduce an elevated pulse, and moisten a dry skin, while one grain doses will irritate both; neither can they believe that Quinine can be used like Calomel, in fevers, in connection with bleeding, and other misnamed *antiphlogistic* remedies. All the generic terms of the Materia Medica and the Practice of Physic, are, in my opinion, great obstacles in the advancement of our profession. We extract blood from the veins, in directly opposite conditions of the system, with benefit, under given circumstances. In the one case, we are said to relieve, by reducing the pulse—and, of course, in the other, we should say, by elevating the pulse; but in fact, the reduction and the elevation, are equally the effect, and not the causes of relief. So with the remedies which are named according to the secretions which they most generally open. The sweating, for example, in fevers, is not the means but the consequence of relief. My first dose of Quinine, is generally followed by greater sweating than the second, and that than the third, because the latter have less of that disorder to counteract, which closed the secretion. But sometimes the nervous disorder is equally counteracted by the Quinine, without any perspiration, instead of which, the urine begins again to flow. In short, as all the visible phenomena of disease, are the consequence of imperceptible disorder, so all the perceptible changes, towards health, are the consequence of the invisible counteracting impressions of the remedy. You perceive I am trying to express my ideas without even using the convenient term of nervous system. For a long time physicians believed that Calomel cured diseases by *salivation*, which exploded opinion, is not a bit more ridiculous, than the belief that it cures fever by *purging*. Speaking in conformity with such opinions, I should say that in large doses, it cures by diaphoresis or diuresis, or both. All that I know about Cholera, is, that some imperceptible disorder, causes the discoverable phenomena of excessive secretion into the intestines, coldness of the surface, &c.

I infer that Calomel corrects that imperceptible disorder, because these discoverable phenomena cease when enough is timely applied.

Hence, I see little or no advantage in external frictions, plasters, heat, &c. which can only tend to counteract one or more visible effects of the invisible disorder—while the Calomel, invisibly counteracting that invisible disorder itself—the whole visible effects must necessarily cease. The Calomel, then, does not cure the Cholera by salivation, nor by catharsis, nor by perspiration, nor by diuresis, but by counteracting that invisible disorder, in which all the visible disorders of the patient depend. The consequent excessive secretion from the intestines, and skin, must be suspended—the suspended secretions of the liver and kidneys must be restored, and all the functions be placed in that equilibrium which they had lost.

In the Campeche Cholera, the progress was so rapid, and the patients so numerous, that bleeding would have rarely been used, had it even been advisable as an auxiliary in the treatment. As a curative, I presume no one has ever thought of trusting to it in the exhausting Cholera. I may see a case in circumstances similar to those which sometimes render bleeding a useful auxiliary in the treatment of hæmorrhage, but am not conscious that any such occurred in Campeche.

I have long since formed and expressed the opinion, that Quinine in large doses, often repeated, would cure the Cholera. The little use which I have made of it in Campeche, confirms that opinion. As my services were entirely gratuitous—the Quinine very scarce and dear—and the sick so numerous, that the flying visits to those who were visited at all, were very few. I could not, of course, trust any conclusive cases to the Quinine alone. For myself, however, and for particular friends, I used five grain pills, whenever a diarrhœal discharge occurred, which was generally suspended by three or four pills. In one case of violent Cholera, where, in five or six hours after a forty grain dose of Calomel, the vomiting and purging returned, a twenty grain dose of Quinine, apparently completed the cure. So in weakly or weakened persons, with cold or sensation of cold, I have added a twenty grain dose to the Calomel forty grains—and have repeated five grains of Quinine, after every watery purging, or every half hour, until the surface was covered with a warm sweat. Should I have an opportunity of seeing the Epidemic, in such a form, that I can limit my services to half a dozen patients daily, I shall be disposed to give a fair trial to large doses of Quinine alone. The treatment by Calomel alone, is so very simple, that I should hereafter say to the public: Have an ounce of Calomel in every house, made up into ten grain pills. As soon as an individual is attacked with watery vomiting or purging, give him four to eight pills, (forty to eighty grains,) according to the violence of the symptoms, and repeat the same quantity after each copious or debilitating discharge from the mouth or anus. Cover the patient with as many blankets as may feel comfortable. Let him take any drink of any temperature that is most agreeable to his taste and stomach, as often as he pleases, but not in such quantities as to provoke vomiting, say in tablespoonsful at a time. Rub his spine well with coarse dry cloths, as long as cramps or vomitings occur. You *may* add red pepper to his ancles, over his stomach, and along his spine, till the physician arrives. To the physician, I should say, when you arrive at a patient thus treated, his vomiting, purging and spasms, will have probably all ceased, heat will have likely returned to his extremities, and his whole surface will as likely be covered with a warm sweat; in short, you will find him convalescing, instead of dying. *But* although vomiting and purging, both may have ceased, and the friends may not, therefore, have given more than one dose of Calomel, you may occasionally find the patient still sinking, his face emaciating, cooling, pulse failing, &c., in short, with all the other symptoms of exhausting Cholera. In this case, the flow of serous fluid into the intestinal tube has not ceased, and is exhausting him as rapidly as an internal flow of blood. In weakly or weakened habits, a small quantity of either extracted from the vessels, will prove fatal to the system, whether retained in or discharged out of the cavities of the body. If this secretion into the intestines be not speedily arrested, your patient will speedily die with his bowels full of water. Repeat, then, the Calomel, every fifteen or twenty minutes, until natural warmth and moisture appear all over the body and extremities. We do not hesitate to give enormous doses of Opium in Colic and Tetanus—why shrink from giving proportionable doses of Calomel in the rapidly fatal Cholera? You will find that while it calms irritation, and checks excessive secretion in one set of organs, it increases action, and opens the deficient secretions in others. The greater the dose, the greater its anticathartic effects.

If Opium is an advisable auxiliary at all in Cholera, it is likely indicated by pain alone. If Calomel alone will cure the Cholera in one case, it should cure the Cholera in all similar cases, without Opium. If the irritation is calmed, and the spasms are counteracted in one case, by large doses of Calomel alone, why not in all cases by the same remedy alone? If it requires a certain quantity of this medicine, to counteract all the symptoms of Cholera, why not give the whole quantity in the shortest possible time? The Opium which I have used as an auxiliary in Cholera, has been more in conformity to the opinion of others, than of myself. It is true, that when the Epidemic began to decline, and common diarrhœas take its place, it was the only medicine that I used, but then it was per anum, and with the injunction that if the discharges should become suspicious, to swallow immediately a large dose of Calomel. To the public I said, in these cases, go to bed as soon as a diarrhœal discharge occurs, and take nothing but toast water, until it ceases, but insert also a pill of opium, in the rectum, after each deposition; and if the discharges be not then speedly checked, or should be accompanied by a sense of debility, take a large dose of Calomel, and receive no food until hunger returns, when you may use rice gruel or arrow root, until you feel entirely well.

I never saw such a people as the Mexicans for feeding the sick. The first question put to the doctor is, not what remedies, but what food the patient must take. Hundreds were killed by stuffing them with soups and gruels, as fast as they were vomited or purged, to prevent the patient dying of debility. I soon became so angry, that the moment I entered a house I shouted "*Si me nombren alimenti a debilidad non receto.*" If you name debility or aliment, I will not prescribe. Twenty four hours in bed, with toast water, were generally sufficient to check these diarrheal *after* dinners of the Cholera.

I was somewhat prepossessed, at the beginning of the Cholera, in favor of external heat and moisture. I now am only in favor of the *trial* of it, for fifteen or twenty minutes, in the shape of a vapor bath, whose heat and moisture is participated by the lungs. On the 4th day, of the Cholera, I ordered the respirable vapor baths at the hospital of San Juan de Dios. The assistants were so pleased with its effects, that they declared in case of being themselves attacked, the vapor bath should be their first remedy—but poor fellows, when the time came, there was no body to administer the bath to them—and they died without even being seen by the superintending physician, who soon followed them to the grave, under the Broussais practice. I had warned him against the falsehoods of this notorious Frenchman—I showed him the soaked surface of the intestinal tube of the first patient who died in the hospital on the 24th June, at midday—he had even seen the recoveries under large doses of Calomel—but when he fell sick, the Spanish-French prejudices and fears overcome him—he sent for the old Spanish and the old French Doctors, and I did not see him, till they had abandoned him, and he was dying. If one could have been amused during such an epidemic, he would have been diverted at the capricious vacillating practice of the Spaniards and the Frenchman, while they continued in the streets—Musk, Assafœtida, Columbo, &c. &c. in succession, or combined. Every day something new, which was abandoned the next, until all were given up in despair. Even the famous *Guaco* became despised for its inertness.

24th September. As the vessel has not yet been despatched, I may as well add such facts and reflections as occur to me, because using a manifold letter writer, however tiresome this communication may be to you, the simultaneous copy will at least be useful to my memory. You are aware, that not above one fifth of the population of Yucatan, is composed of persons entirely white. The native Indians, called the Mayas, are not only a distinct race, from those of the United States, but also differ as widely from the other Indians of Mexico, as the French from the Russians. They are indolent and ignorant—humble and good natured, and are the laboring citizens or peasantry of the country. During the first week very few indeed were the persons of entirely white blood, attacked by the Cholera in Campeche. In one village, the mortality was so completely limited to Indian blood, that the natives imagined they were poisoned by the whites, and were on the point of revenging themselves by force, when luckily the death of a few white inhabitants, enabled the Alcalde to convince the Mayas of their error. Negros and their mixtures form a very small part of the population, and were as great sufferers as the Indians—possibly greater—in proportion to their numbers. Even the different shades of Indian and Negro blood, seemed to invite the disease in the ratio of their darkness. All the servants of large families were, in some instances, swept off without exception, while the white members were not even attacked.

I perceive that you are strongly opposed to the doctrine of contagion in Cholera, and so am I, but there are many circumstances which tend to show that the poison is transportable. Have we not facts to show that a vessel arriving at a port in the United States from the West Indies, with a healthy crew and passengers, may, nevertheless, contain enough poisonous atmosphere in her hold to produce the Yellow Fever in persons employed to discharge the cargo? In October last, during the Cholera of New Orleans, vessels came thence to the ports of Yucatan, without bringing the disease, although in November last, at Liral some sailors actually died on board with the Cholera. In March last, vessels came from Havana, and although aided by the trade wind, did not bring us the Cholera. But in June, a vessel came from Tampico, with sick on board, against the prevailing winds, quarantine regulations were eluded and in a few days afterwards we had Cholera in the suburbs of Saint Roman—the parish where the crew and effects were landed. The Cholera has ever since continued spreading from Campeche through the

Peninsula towards all points of the compass, and of course, to the Eastward, in the very teeth of the trade wind. The Villa del Carmen, however, about forty leagues to the S. W. *on an Island*, at the Laguna de Terminos, being enabled by its position, to cut off rigorously all communication, has hitherto escaped, although the Cholera has extended to the plantations on the adjacent shore of the main land. Some plantations to which no person has been admitted, have escaped the desolation of others around them, as we learn by persons who have left these still healthy positions, on the express condition of not returning until the Cholera should entirely cease in all places which they may visit during their exile. As very little travelling is done in Yucatan, and that principally by means of passports, it is very easy to trace the transportation of the disease from one point to another, and I therefore hope that when it shall have ceased in this Peninsula, we shall be in possession of facts enough to decide the question of the transportability of the Cholera poison. Whatever be the result, whether in favor or contrary to your present opinions relative to the origin and propagation of Cholera, I doubt not that you will be equally contented with the acquisition of fresh facts, relative to this mysterious epidemic.

You are doubtless aware, that the whole Peninsula of Yucatan is proverbial for its healthiness, owing, no doubt, to the peculiar dryness of its soil and atmosphere, with the wonderful uniformity of temperature, which is common to most shores of the miscalled torrid zone. Indeed every part of the coasts of tropical seas which has no marshes nor mountains to poison or chill its atmosphere, presents in its dry and equable temperature the means of health and enjoyment, which cannot be found in any part of the misnamed temperate but properly variable zone.

Driven into the slandered torrid zone, to find in its equable temperature the natural remedy for disease, induced by the variable temperature, which is the principal evil of all our existing States, and common to every part of the miscalled temperate zone, yet amidst all the numerous natural enjoyments of this delightful climate, I daily become more and more acquainted with the innumerable unnatural sufferings resulting from the character of its inhabitants and of its government. As the wonderful uniformity of temperature, the extraordinary varieties of vegetation, and the consequent natural superiority for physical health and pleasure, enjoyed by the dry shores of a tropical sea, can only be adequately estimated by natives of the changeable north, after personal experiment, so the wonderful suavity of the governments, the peculiar characteristics of the people, and the consequent social superiority for intellectual health and happiness enjoyed by the United States of North America, can only be thoroughly appreciated by its own citizens after long residence abroad. Hence, having ascertained that the dry winter, the wet summer, the trade winds, the daily sea breeze, the nightly land breezes, and above all, the uniform temperature of this climate, extends several degrees north of the Tropic of Cancer, and consequently into our own territory, you will pardon my enthusiasm in repeating the conviction, that southern or tropical Florida, in climate, position, and government, combines more natural, social, and political advantages than any other portion of the world.

With these views of the superiority of climate and productions of the tropics, and of the government and people of the United States, and the peculiar position of the peninsula of Florida, giving it the advantages of both, the speedy population, and planting of its tropical extremity, is of immense national importance both to our healthy and sickly citizens. When remote from marshes which send noxious moisture into the air, or from mountains which disturb its otherwise equable temperature, its tropical seashore is the most healthy residence, not only for our citizens who are laboring under pulmonary disorder, but also for those who are suffering under hepatic disease. The common belief of the general insalubrity of the torrid zone, is a popular error created by the resorts and the reports of war and trade; the spots most frequented by commerce and of course most liable to be the seats of war, are generally surrounded by the local circumstances which would generate disease in every climate of the globe under equal conditions of mental anxiety and corporeal exposure. But the very city from which I am writing—the whole peninsula of Yucatan—is proverbial for the dryness, healthiness of its atmosphere. The surface, which is level, calcareous and continually swept by the trade winds, has never been traversed by any epidemic but the present unaccountable Cholera.

Having read in the May number of the Philadelphia Journal of the Medical Sciences, the review of Johnson on the "change of air," I was pleased with the addition to the remarks of the author on the climate of Italy, and especially with the beginning of the "momentous enquiry to select the situations best adapted during the cooler months for those who have a tendency to or are laboring under pulmonary affections," to which might be added, and for the hepatic disorders equally aggravated by sudden vicissitudes of temperature, which are as common in Louisiana as in Maine. To the editor of that Journal, and to yourself, I therefore point out Cape Florida as the most eligible winter retreat, for our citizens suffering under diseases of variable temperatures, and through both your Journals, I would challenge my professional brethren, to show any situation out of tropical Florida, which combines as many advantages for such persons, when travelling in pursuit of health and enjoyment.

26*th Sept.*—Although well aware of the great influence of the mind on the body, I never conceived it to be so powerful as it has shown itself in the Cholera. One death was generally followed by several others among the sensitive community—not of relations nor house-mates only, for the acquaintances or friends were at least as frequently carried off, however different their temperaments or distant their residence. In the same family the cases were not proportioned to the debility of the body, but to the weakness of the mind. Keeping up myself always a cheerful countenance and even jocose tongue, when I found an individual whom neither my presence nor words would animate, I marked him as a speedy victim of the Cholera, in which medicines would be of no avail. In some instances I refused to visit such, assigning my reason to those relatives who exhibited more mental valor. On the othe hand, the courage of one individual would save the rest. Col. Jose Mendez, former Judge of the District, made his will and died without any symptoms of Cholera, like the wasting of a candle. His sister, a female devotee, went off with hysterics on the same day, not on his account—but to join a female friend whom the Cholera had just destroyed. The brother, Pedro Mendez, Secretary to our Cholera Board of Health, always shuddered at the name of the disease before it reached the Peninsula—shut himself up when it arrived—and offered to serve me on his knees all his life, if I would save it. I told him that with his silly fears medicines could not operate; and when called to him six hours after his attack at midnight, found him cold and pulseless, and ordered him to dispose himself with the Notary and Priest. Dr. Santiago Mendez, another brother 35 years old, would have followed his two brothers and sister, had not his wife possessed the character of a Roman.—Neither of the dead, you observe, had suffered from grief for each other, but he was suffering sorrow for the three with a melancholy presentiment that all their large family must sink under the epidemic from similarity of constitution. While another brother, a Priest, and a sister were convalescing in the same house, with all the servants on sick beds, Mrs. Mendez was so much engrossed in their cases, being the only attendant, that she did not observe her oldest boy was sick during night, until she found him dying in the morning. She concealed her distress, and hurried off her husband to a room in the college of San Jose—and I accompanied him as I considered his life worth more than the lives of a hundred others. He was a liberal patriot, and what is still rarer in Mexico, an honest man. He had refused the nomination for Governor, and was elected vice Governor without his knowledge. In short, he is a man who I believe will be of more moral service to Yucatan, than any other ten inhabitants of the Peninsula. With the view of seeing him often, what little rest or nourishment I took, I sought at the College. About 2 A. M., however, on the second day he woke from a frightful dream, with a noise which startled me. The fright was immediately followed by a discharge from his bowels, which left him fainting in the chair—indeed so lifeless did he appear, that the Priests, among which was a brother, fled—and I was left alone. I threw part of a bottle of wine in his face, shouted for help—carried him to bed—gave him 40 grains of Calomel—sent for his wife, went for lively friends—and without details—by mid-day he was out of danger—but without the words and deeds of his wife, he would have surely died. The bells had been tolling "rogativas" for 13 days, when the thought occurred to this heroic woman, of changing their mournful tones to the lively notes of a Te Deum.

Ascertaining that a less number of deaths had occurred the day before, on Saturday, than on Friday, I immediately called together the Priests, visited the Commanding General, and at noon the despairing population of Campeche was delighted at the surprizing but merry ringing of the bells, and firing of the cannon, which continued during the whole afternoon. Of its wonderful effects in arresting the Epidemic, you will be able to judge when I obtain the official list of burials —the ringing of the bells was repeated every noon, and at the end of the week I declared that the Cholera had ceased as an epidemic, but the authorities were unwilling to give clean bills of health till a week afterwards.

28*th Sept.*—A letter from the Alcalde of Cibalchem, a village 30 leagues to the South of East from Campeche—tells me that on the 18th of August, the Cholera entered so mildly that in 12 days they counted but 5 or 6 deaths to more than 30 convalescents—but that on the 2d inst, it attacked with such violence as to carry to the grave 15 to 20 bodies daily. None of the principal inhabitants however suffered until the 11th, when Dr. M. Privero was carried off by vomiting and purging, and the 14th when Dr. J. de la Cruz followed him with diarrhea alone, both wealthy individuals who endured but 9 or 10 hours. During the first fifteen days, the persons attacked nearly all got well, under any kind of treatment, but during the next fifteen days, up to the 19th inst., the date of the letter to me—out of every twenty attacked, not more than one, and at most two, escaped under every domestic care, (as there are no physicians there) for although the symptoms were not alarming, not one lasted 24 hours. Thus says the Alcalde, requesting from me medicines and advice. So you perceive that in Cibalchem, the epidemic pursued a course directly opposite to its career in Campeche, where in the first fortnight its ravages were dreadful, and in the last fortnight comparatively trifling. To-day we are beginning to be alarmed anew, as two cases were reported day before yesterday, and two more have entered the hospital this morning. As the vessel is to sail for New Orleans this afternoon, it is doubtful whether I shall be able to send you any details.

I have the honor to be, sir,

Very respectfully, your ob't. serv't.

HENRY PERRINE.

Dr. Drake.

